# Effect of Rubber Granulate Content on the Compressive Strength of Concrete for Industrial Vibration-Isolating Floors

**DOI:** 10.3390/ma18133134

**Published:** 2025-07-02

**Authors:** Maciej Gruszczyński, Alicja Kowalska-Koczwara, Tadeusz Tatara

**Affiliations:** Faculty of Civil Engineering, Cracow University of Technology, 31-155 Kraków, Poland; maciej.gruszczynski@pk.edu.pl (M.G.); ttatara@pk.edu.pl (T.T.)

**Keywords:** rubber granulate, vibration isolation, industrial floors, concrete subbase, compressive strength, recycled materials

## Abstract

Ensuring vibration and impact isolation is crucial in industrial flooring design, especially where vibroacoustic comfort is a priority. Excessive vibrations can negatively affect sensitive equipment, structural durability, and personnel comfort. With the rise of automation and high-precision processes, effective vibration control in floor systems is increasingly important. Traditional solutions like elastomer pads, rubber mats, or floating floors often have high installation costs, complex construction, and long-term degradation. Therefore, there is growing interest in integrated, durable alternatives that can be incorporated directly into concrete structures. One such approach uses rubber granulates from recycled tires as a modifying additive in cementitious composites. This can improve damping, enhance impact energy absorption, and reduce the need for external insulating layers. However, adding rubber particles to concrete may affect its compressive strength, a key design parameter. This article presents experimental research on concrete and mortar mixtures modified with rubber granulates for vibration-isolating industrial floor systems. The proposed solution combines a conventional concrete subbase with a rubber-enhanced mortar layer, forming a composite system to mitigate vibration transmission. Laboratory tests and real-scale verification under industrial conditions showed that the slab with hybrid EPDM/SBR rubber granulate mortar achieved the highest vibration-damping efficiency, reducing vertical acceleration by 58.6% compared to the reference slab. The EPDM-only mortar also showed a significant reduction of 45.5%.

## 1. Introduction

Industrial floors are critical structural components in manufacturing, storage, logistics, and public utility facilities. Their performance under mechanical, chemical, and environmental loads directly impacts the operational efficiency, safety, and longevity of the built environment. Maintaining floor cleanliness in agri-food buildings is crucial to prevent product contamination and identify critical issues using tools like spectrophotometric analysis [1]. The authors of [2] highlight the importance of floor design in citrus juice and essential oil manufacturing, where floors face corrosion from hot oils and chemicals, thermal shock, and damage from heavy machinery and traffic. Concrete floors, in particular, are widely used due to their high compressive strength, load-bearing capacity, and cost-effectiveness [3,4,5]. Other flooring systems, such as epoxy and polyurethane coatings, are applied where chemical resistance, easy cleaning, or aesthetics are required, while resin floors are preferred for high-abrasion environments [6,7]. Ceramic and wooden floorings serve specialized applications in food processing or sports facilities, respectively, where hygiene or user comfort are priorities [8].

A typical industrial concrete floor is a multilayered system, comprising a stabilized soil subgrade, a load-distributing subbase, a slip membrane to control stress transfer, a reinforced concrete slab, and a screed or surface coating that ensures resistance to abrasion and chemicals [4,9]. These structures must meet numerous design criteria, including load capacity, dimensional stability, flatness, abrasion resistance, slip resistance, thermal and shrinkage deformation tolerance, frost resistance, and ease of maintenance [3,5,10].

Despite established design practices, failures in industrial floor systems remain common. These include early-age cracking, surface scaling, curling, delamination, or localized subsidence, typically resulting from errors in structural detailing, material selection, or execution [11,12]. Excessive or dynamic loading, foundation settlements, chemical exposure, and lack of expansion joints can exacerbate deterioration. Moreover, floors in industrial environments are subject to evolving operational demands: changes in equipment layout, increases in dynamic or point loads, and alterations in temperature gradients across the slab [13].

In recent years, particular attention has been paid to the vibroacoustic performance of industrial floors. Vibrations originating from machinery, forklift traffic, or adjacent operations can lead to user discomfort, affect the performance of precision equipment, and accelerate structural fatigue [14,15]. While traditional solutions for vibration isolation include rubber or elastomer mats, floating slabs, or resilient padding systems, these approaches often introduce executional complexity, increase costs, and degrade over time under load or chemical influence [16].

As a response, integrated solutions have been proposed to incorporate damping capacity directly into concrete or mortar compositions. One promising approach involves the use of rubber granulate—typically obtained from recycled tires—as a modifying additive to cementitious materials [17]. Rubber particles can improve energy absorption, reduce stiffness, and enhance damping properties of concrete, making it suitable for low-frequency vibration isolation [18,19]. However, incorporating rubber into concrete typically leads to a reduction in compressive strength and changes in modulus of elasticity, which pose challenges for structural applications [20,21].

This study investigates an innovative industrial floor system consisting of a standard concrete subbase and a vibration-isolating mortar layer modified with rubber granulate.

The article presents a novel approach to the design and evaluation of industrial floors with integrated vibration isolation, using rubber granulate-modified cementitious materials. The key steps and findings are outlined below:

- Context and motivation—Increasing importance is being attributed to vibroacoustic comfort in industrial environments, especially in facilities with sensitive equipment or automated processes. Traditional vibration-isolating solutions (e.g., elastomer mats) are often expensive, difficult to install, and prone to degradation.
- Integrated damping layer concept—A composite floor system is proposed, where a 5 cm-thick damping layer made from rubber granulate-enhanced mortar is cast beneath a standard reinforced concrete slab. This layer replaces conventional insulating materials with a monolithic, construction-friendly solution.
- Material selection and mix design—Rubber granulates (EPDM and SBR) were used to partially or fully replace natural aggregate in subbase concrete and mortars. Mixtures were tested for workability, consistency over time, and compressive strength to ensure suitability for structural use.
- Laboratory and pilot-scale testing—Both lab-scale mechanical tests and in-situ dynamic testing of full-size slabs under controlled vibration (using the Mark IV vibroseis system) were performed. Three floor variants were compared: reference (no damping layer), EPDM-only, and EPDM + SBR hybrid.
- Effectiveness of vibration isolation—Significant reduction in vertical accelerations (Z-direction) was observed: 45.5% for the EPDM layer and 58.6% for the EPDM + SBR hybrid layer.
- Practical implications—The proposed solution enables vibration isolation to be built directly into concrete floor systems using recyclable materials. The mixes showed stable consistency, good self-compacting behavior, and compatibility with standard production methods, suggesting strong potential for real-world application.

The research focuses on evaluating the mechanical and vibroisolation performance of mixtures containing three types of granulates: SBR (styrene-butadiene rubber), EPDM (ethylene-propylene-diene monomer rubber), and a 50/50 SBR–EPDM blend. The proposed design seeks to replace conventional elastomer mats with an integrated damping layer in the screed. The performance of the system was evaluated through laboratory-scale mechanical and dynamic testing, followed by a pilot-scale trial under industrial conditions. This approach supports the development of multifunctional, sustainable industrial floors with enhanced vibroacoustic comfort and optimized structural performance.

## 2. Materials and Methods

### 2.1. Reference Mixture

In the first stage of the project, based on the analysis of the current standard [21], preliminary assumptions were made regarding the basic technical properties of the subbase concrete mixtures, namely:

- compressive strength class: C8/10 to C16/20;
- target layer thickness: 15 to 40 mm, which determined the maximum aggregate size (D_max_ = 8 mm);
- consistency class: S4/S5 (i.e., slump ≥ 200 mm), maintained for at least 60 min, with the required self-compacting ability of the mix.

Commonly available natural aggregates of 0/2 mm and 2/8 mm fractions were selected for the concrete mixtures.

Ground granulated blast furnace cement CEM III/A 42.5 N was used as the binder. This cement was selected due to its high strength combined with low permeability of the cement paste, limited shrinkage, and favorable heat of hydration characteristics, which collectively ensure the durability of the concrete structure.

To ensure proper workability, pumpability of the mix, and compliance with the standard requirement for minimum fine particle content (i.e., below 0.125 mm) per cubic meter of concrete, a Type II mineral additive—siliceous fly ash category A (according to PN-EN 450-1) [22]—was incorporated into the mix design.

A rheological admixture based on polycarboxylate ether was selected for the concrete mixtures. It proved fully compatible with both the selected cement and fly ash, ensuring that the required consistency of the mix was maintained for a minimum of 60 min.

**Figure 1:**
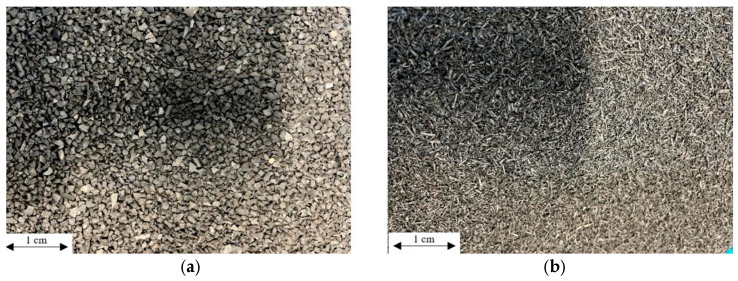
Vibroinsulating additives: EPDM granulates (**a**) and SBR granulates (**b**).

− To improve the vibration isolation capacity of the concrete layers, the following materials were selected as modifying additives (Figure 1a,b): rubber EPDM granulates (1/3 mm fraction) and SBR granulates (0.8/2 mm fraction).

As a part of the project, sieve analyses of the aggregates were carried out, followed by the determination of the mineral mix composition, ensuring maximum aggregate packing density. This was achieved using an iterative approximation method. The outcome of the procedure was the identification of optimal weight proportions of sand and gravel to achieve the densest possible granular skeleton, namely, sand 0/2:gravel 2/8 = 2:1 (by weight). After completing the iterative optimization of the aggregate composition, the final formulation of the subbase concrete mix was established (see Table 1).

To verify the properties of the designed concrete mix, a trial batch was prepared in the accredited laboratory of the Department of Building Materials Engineering at Cracow University of Technology. The following properties of the fresh concrete mix were tested:

- consistency using the slump cone method (in accordance with [23]) and flow time t_500_ (in accordance with [24]);
- fresh concrete density (according to [25]).

Test specimens were cast from the reference mix in the form of six cubes with 100 mm edge length for compressive strength testing after 7 and 28 days. The specimens were cured in water at a temperature of 20 °C.

The properties of the reference subbase concrete mix are summarized in Table 2.

The results obtained from the fresh concrete tests confirmed full compliance with the preliminary design assumptions. The trial batch also demonstrated the compatibility of the polycarboxylate-based rheological admixture (PCC) with the selected binder system (cement + fly ash). An increase in flowability of the mix was observed during the first 60 min after mixing. Furthermore, the concrete exhibited very good workability and showed no tendency toward segregation of its components.

The compressive strength test results after 7 and 28 days of curing are presented in Table 3.

The compressive strength obtained exceeded the required values with a significant safety margin. This was intentional, as the negative impact of vibration-isolating additives on the mechanical strength of concrete was anticipated. Therefore, the reference mix was deliberately designed with a strength reserve.

It should also be noted that, due to the use of a blended binder system (blast furnace cement and fly ash), strength gains are expected to continue up to 90 days of curing.

Based on the rheological and mechanical test results, the reference subbase concrete composition was selected as the basis for further modification using vibration-isolating additives. These additives were used to replace 30%, 60%, and 100% of the aggregate fraction by volume. The consistency of the modified mixes was controlled by adjusting the dosage of the PCC rheological admixture in order to achieve the target consistency level (i.e., slump greater than 200 mm).

Trial batches were prepared at laboratory scale using the following materials: rubber EPDM granulates (1/3 mm fraction) and SBR granulates (0.8/2 mm fraction).

The properties of the rubber-modified concrete mixtures are presented in Table 4. For practical purposes, consistency was measured using the slump test method after 10 and 60 min from mixing.

The compressive strength results of subbase concrete mixes modified with vibration-isolating additives are presented in Table 5.

### 2.2. Self-Compacting Subbase Concrete Mixtures

Based on the results obtained for rheological properties and compressive strength, the following additive compositions were selected for further investigation: EPDM rubber granulates (1/3 mm fraction), SBR rubber granulates (0.8/2 mm fraction), and a 50/50 blend of EPDM (1/3 mm) and SBR (0.8/2 mm) rubber granulates.

For all selected variants, the replacement level of natural aggregate with rubber granulates was set at 100% in order to maximize the damping effect. As a consequence of the complete replacement of conventional aggregate with rubber additives, it was necessary to optimize the dosage of fly ash to ensure mix stability and achieve self-compacting properties.

The formulations of these concrete mixes are presented in Table 6.

**Figure 2:**
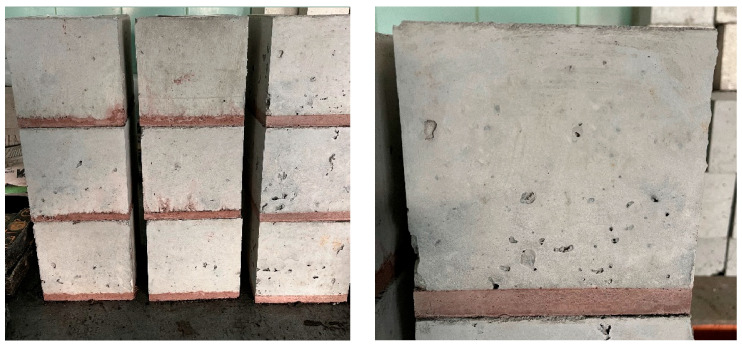
View of composite specimens—15 mm damping subbase layer (colored red) and 135 mm C35/45 XM2 industrial topping layer.

To verify the properties of the proposed subbase concrete mixes, trial batches were prepared. The self-compacting ability of the mixes was evaluated at 10 and 60 min after mixing. Test specimens were cast to assess compressive strength after 7 and 28 days of curing. Additionally, composite samples were prepared in the form of 150 mm cubes, cast in two layers: the first layer (15 mm thick) consisted of the rubber-modified subbase mix, and the second layer (135 mm thick) was added after 24 h using a standard industrial-grade concrete mix of class C35/45 XM2 in accordance with [19] (Figure 2).

For better visual distinction, the subbase mix was colored red by adding 1% by mass of a standard concrete pigment.

Detailed results of the consistency tests of the vibration-isolating mixes, as well as the compressive strength measurements, are summarized in Table 7.

In the next stage of the research, the damping efficiency of the isolating layer was evaluated. This was performed using composite cube specimens with edge lengths of 150 mm. Each specimen consisted of a 15 mm-thick layer made of the vibration-isolating mix (colored red for technical purposes) and a 135 mm-thick overlay made of a standard industrial concrete of class C35/45 XM2 (Figure 2).

The vibration-damping effectiveness was assessed using a type N Schmidt rebound hammer manufactured by Proceq (Schwerzenbach, Switzerland). The tests were carried out on composite specimens placed in a universal testing machine, which applied an axial compressive stress of approximately 6 N/mm^2^. The rebound number was measured on the surface of the vibration-isolating layer.

For each of the three specimens in a series, nine rebound values were recorded. Values deviating by more than 20% from the mean were discarded. Detailed results of the rebound number tests for each mix series are presented in Table 8.

The obtained sclerometric test results for concrete layers with the addition of rubber granulates (EPDM and SBR) were in the range of 25–29 and were about 40% lower than in the case of the reference concrete, for which the average rebound number was 41. This allows us to conclude about the high ability of these materials to dampen vibrations.

**Figure 3:**
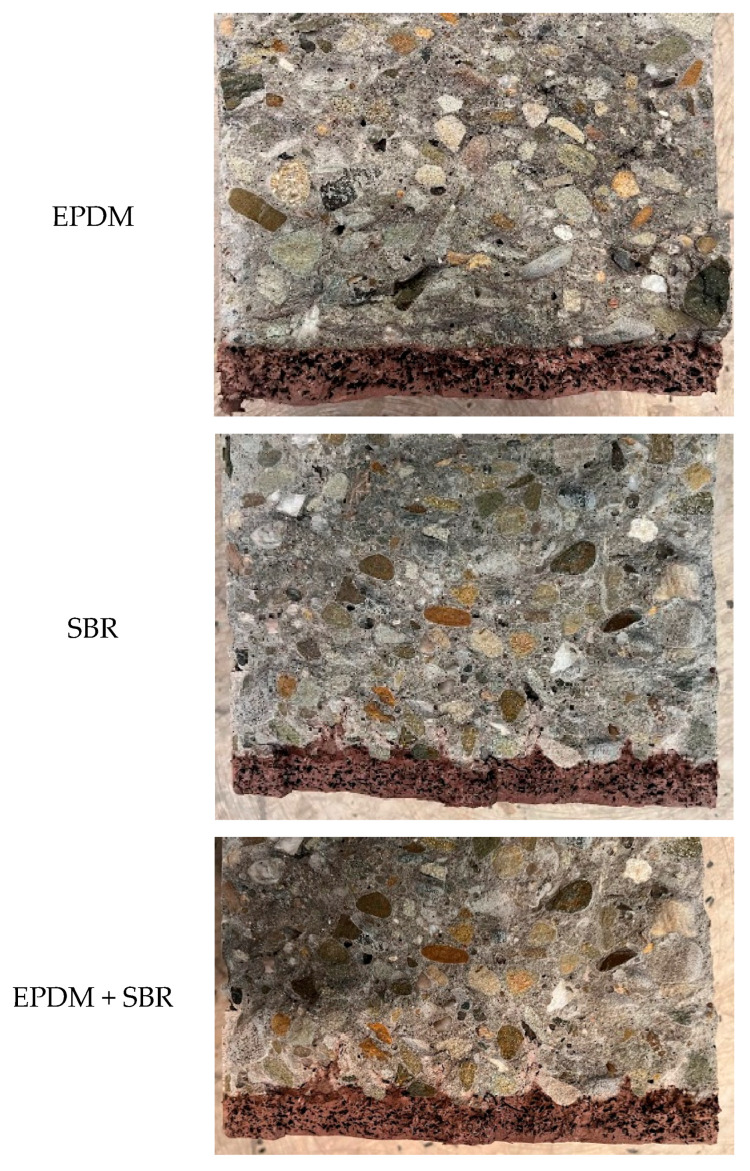
View of split composite specimens (damping layer colored red).

After the Schmidt hammer test, the composite specimens were split to assess the uniformity of rubber granulate distribution within the damping layer (Figure 3).

The results of the tests conducted confirmed the uniform distribution of rubber granules in the samples. The results of the analysis of the photographs of the cross-sections of concrete layers modified with the addition of rubber granulates clearly show their homogeneity and lack of delamination, which should be considered a correct result.

## 3. Results

### 3.1. Results of Material Property Tests of Concrete Mixtures

To verify the suitability of the developed concrete and mortar mixes, trial batches were produced at a standard ready-mix concrete plant. As a first step, the surface concrete of class C35/45 XM2 S4/S5, according to [21], was tested. The mix composition is presented in Table 9.

After batching, the concrete mix was loaded into a truck-mounted mixer, and its consistency was measured using the slump cone method in accordance with [23], exactly 10 min after the first contact between cement and water. The measured slump of 250 mm corresponds to consistency class S5 under [21], which meets the project requirements.

Following the consistency test, the mix was subjected to a pumpability test using a 42 m boom concrete pump (CIFA brand). The field tests confirmed full pumpability of the C35/45 XM2 concrete.

After the pumping trial, the concrete mix was transported to the laboratory where, 60 min after water–cement contact, consistency was remeasured using the slump test. Additional tests were carried out to determine air content, and compressive strength specimens were prepared.

The measured air content of the concrete mix was 5.4%, and the slump value after 60 min (from the first contact between cement and water) reached 260 mm. These results fully meet the project requirements as well as the EN 206 standard for consistency class S5.

The prepared specimens were stored and cured in accordance with [23]. Compressive strength tests of the C35/45 XM2 concrete were performed according to [26] after 7, 28, 56, and 90 days of curing. Detailed compressive strength results are presented in Table 10.

The obtained compressive strength values exceeded the project requirements, confirming that the designed concrete meets the minimum strength class of C35/45.

Analogous full-scale trials were performed for the lean subbase concrete. The concrete mix was delivered from the ready-mix plant (RMC), where a pumping test was conducted, and the consistency was assessed 10 min after the first contact between cement and water. The mix was then transported to the laboratory, where its rheological properties were evaluated (slump test and flow spread), followed by the casting of cube specimens for compressive strength testing.

The rheological properties of the subbase concrete mix are summarized in Table 11.

The obtained slump and flow values classify the mix as consistency class S5 according to EN 206, which meets the design requirements.

The prepared specimens were stored and cured following [23]. Compressive strength tests of the lean subbase concrete were carried out using a compression testing machine by [26] after 7, 28, 56, and 90 days of curing. Detailed compressive strength results are presented in Table 12.

The achieved compressive strength values allow the concrete to be classified as strength class C16/20 under [21].

For the final verification of the vibration-isolating mortars, as mentioned in Section 2 of this paper, the following additives were selected as optimal: EPDM rubber granulates (1/3 mm fraction) and a hybrid blend of SBR (0.8/2 mm) and EPDM (1/3 mm) rubber granulates in a 50/50% ratio by weight.

To facilitate the identification of these layers within the floor system, the mortars were colored red using a dedicated pigment at a dosage of 0.2% by mass of cement.

The compositions of the optimized mortar mixes with EPDM and SBR rubber granulates are presented in Table 13.

**Figure 4:**
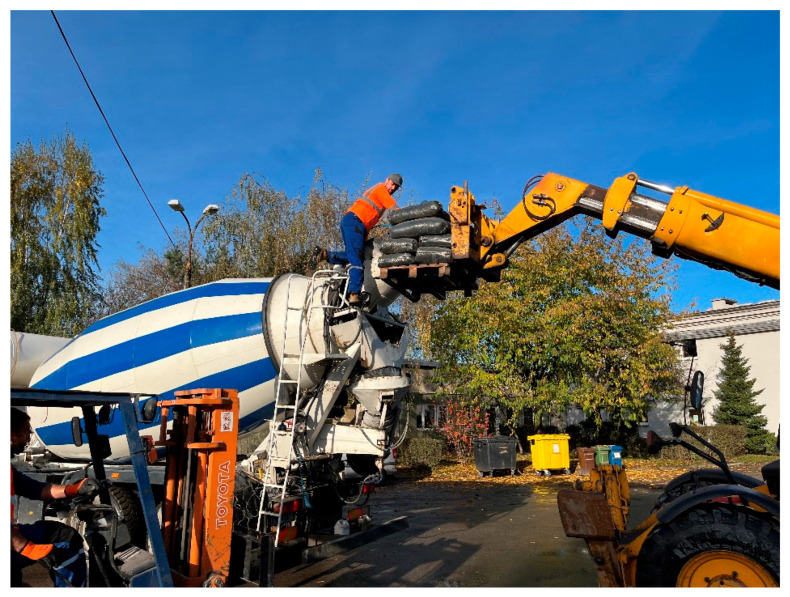
Dosing of EPDM rubber granulates into the drum of the truck-mounted concrete mixer.

As in the case of the surface and lean concrete mixes, the vibration-isolating mortars were produced at a ready-mix concrete plant. Due to technological constraints, the SBR and EPDM rubber granulates were dosed directly into the mixing drum of the truck-mounted concrete mixer (Figure 4).

After the granulates were added to the truck mixer drum, the mortar components were mixed at high speed for at least 5 min until full homogenization was achieved. A sample of the mortar was then taken to evaluate flow diameter at 10 and 60 min from the moment of first contact between cement and water. Test specimens were also cast in the form of 4 × 4 × 16 cm prisms and 10 × 10 × 10 cm cubes for compressive strength testing after 7 and 28 days of standard curing.

The properties of the vibration-isolating mortars containing EPDM and SBR rubber granulates, including flow diameter and compressive strength after 7 and 28 days, are presented in Table 14.

The obtained results for rheological properties and compressive strength are consistent with the design assumptions and confirm the suitability of the tested mixes for use in the construction of a practical vibration-isolating floor system.

### 3.2. Results of In-Situ Dynamic Testing

After verifying the suitability of the designed concrete and mortar mix compositions with respect to the project requirements, a pilot-scale vibration-isolating floor model was constructed.

The test surfaces were prepared on a designated test site (mock-up area) under the following assumptions: the structure was placed on compacted subgrade soil, and standard slab dimensions were used (floor sections measuring 4 × 5 m, separated by expansion joints).

**Figure 5:**
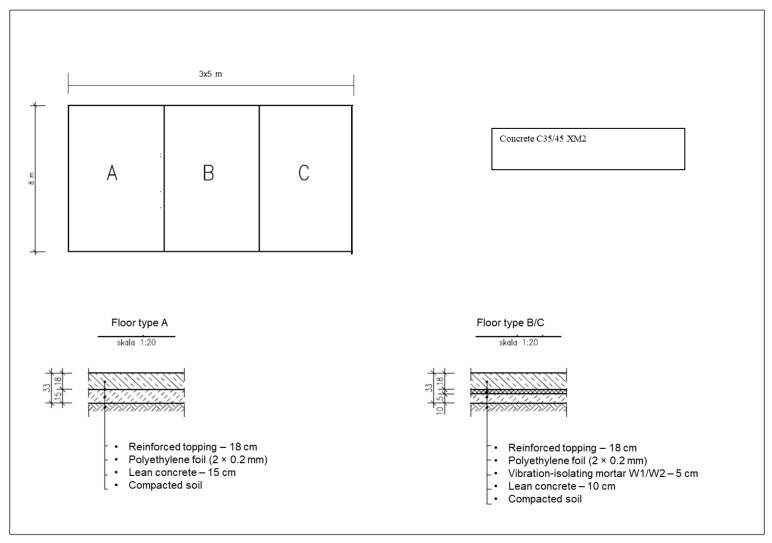
Layer arrangement of floor structures—A: standard floor (without vibration-isolating layer), B: hybrid vibration-isolating mortar with EPDM and SBR granulates (50/50%), 5 cm thick, C: vibration-isolating mortar with EPDM granulate, 5 cm thick.

Details of the floor system model are shown in Figure 5.

To evaluate the vibration isolation performance of the constructed floor slabs, a series of dynamic in-situ tests were carried out using the Mark IV vibroseis system. The excitation was performed in the form of frequency sweeps ranging from 6 to 100 Hz, each lasting 1 min, with the output force set to 80% of the device’s nominal capacity.

The primary objective of the tests was to identify the dynamic response of each floor configuration (A, B, C), the potential resonance zones, and the differences in amplitude transmission.

The test setup included the Mark IV vibroseis placed on the ground in three positions between slabs, and accelerometers in three orthogonal directions were used.

**Figure 6:**
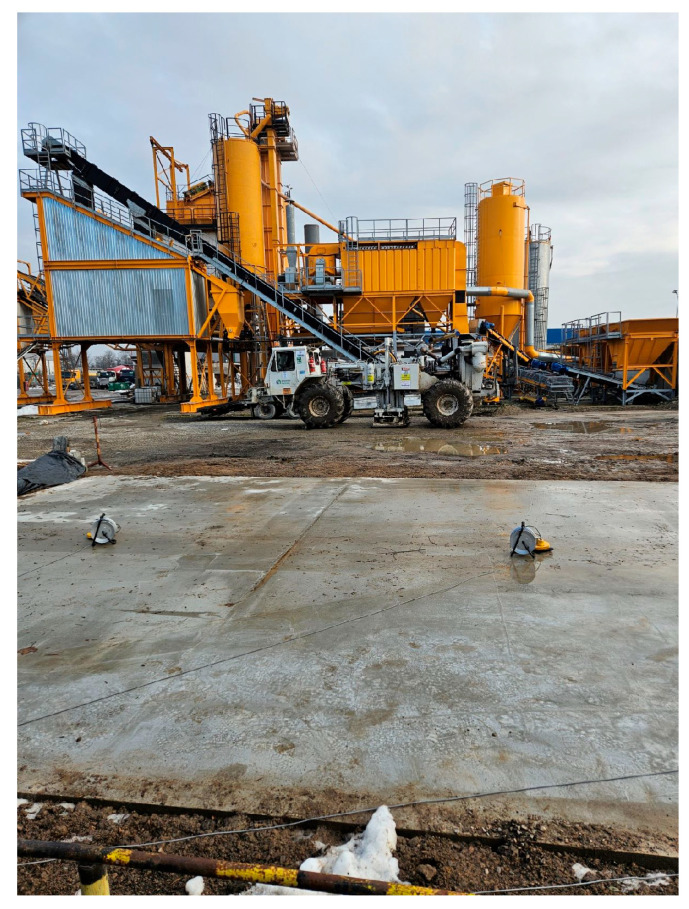
In-situ test setup: Mark IV vibroseis positioned on the ground between the slabs; triaxial accelerometers placed in the center of each floor panel (A, B, C).

The test layout is shown in Figure 6, where the location of the vibroseis and the measurement points are shown.

To further assess the vibration transmission characteristics of the tested slabs, acceleration time histories in the vertical (Z) direction at the center of each slab (A, B, and C) are shown. The excitation was performed using frequency sweeps in the range of 6–100 Hz, with each sweep lasting 60 s and applied at 80% of the rated power of the Mark IV vibroseis.

The analysis focused on the *Z*-axis component, as this direction typically dominates in vibration exposure scenarios affecting building occupants. Vertical vibrations are more efficiently transmitted through structural elements, particularly floor slabs, and tend to generate higher perceptible amplitudes. This is of special significance in terms of human comfort and health, since vertical excitation has been shown to more directly affect internal organs, postural balance, and physiological well-being than lateral motion.

**Figure 7:**
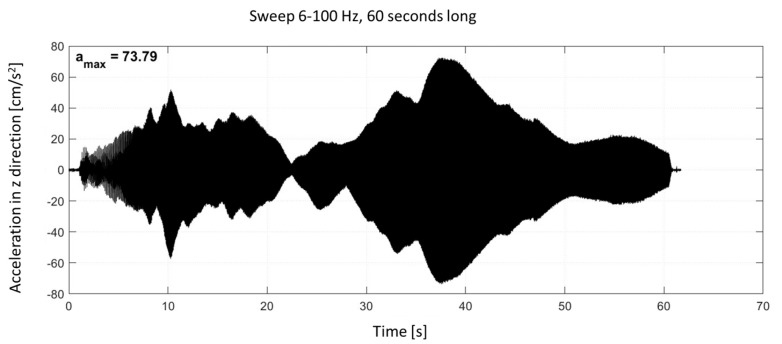
Time-domain acceleration signal in the vertical (Z) direction recorded at the center of slab A during sweep excitation (6–100 Hz, 60 s).

The recorded time-domain signals reflect the complex dynamic response of each slab configuration to the frequency-sweep excitation (see Figure 7), which involves a continuous change in frequency over time rather than a single harmonic input. This type of excitation allows for a broader assessment of the system’s behavior across the entire tested frequency range. Comparing the amplitude levels in the recorded signals enables direct evaluation of the effectiveness of the vibration-isolating layers in reducing vertical vibrations.

In Table 15, the maximum acceleration values (a_max_) recorded in the X, Y, and Z directions are presented for three floor configurations: (A) reference slab without any isolating layer, (B) slab with a hybrid EPDM/SBR rubber granulate mortar, and (C) slab with EPDM rubber granulate mortar.

Each floor type was tested under identical sweep excitation conditions (6–100 Hz, 60 s), and measurements were taken using accelerometers placed at the slab centers. The final row summarizes the mean acceleration values across the three measurement repetitions for each direction and floor configuration.

**Figure 8:**
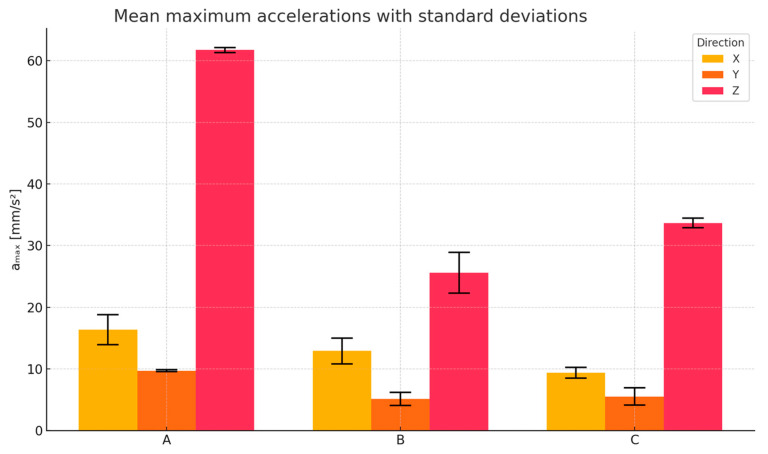
Comparison between the reference floor and the floors with granulate: A—reference, B—EPDM + SBR, C—EPDM.

To compare the vibration response of the three tested floor systems, the mean maximum acceleration values (a_max_) recorded in the X, Y, and Z directions were analyzed. The results, obtained from three repetitions of each measurement, are presented in Figure 8.

Particular attention was given to the *Z*-axis component, which is most relevant to human comfort and health, as previously discussed. The vertical direction often exhibits the highest transmitted amplitudes due to the direct load path through floor systems.

The comparison reveals clear differences in vibration transmission depending on the applied isolating layer. The EPDM + SBR hybrid mortar demonstrates the most significant amplitude reduction in all directions, with a particularly noticeable decrease in the *Z*-axis (58.6%). The EPDM-only mortar also reduces vibrations compared to the reference slab (45.5%), though to a lesser extent.

## 4. Discussion

The conducted experimental study demonstrates that incorporating rubber granulates into cementitious mixtures effectively reduces vibration transmission in industrial floor systems. Among the tested configurations, the hybrid EPDM + SBR mortar exhibited the most significant vibration attenuation, particularly in the vertical (Z) direction, which is critical concerning human comfort and health. Compared to the reference slab, the average maximum acceleration in the Z direction (a_max_) was reduced by over 58%, confirming the efficacy of the damping layer. The EPDM-only mortar also contributed to a measurable decrease in transmitted vibrations, with a_max_ reduced by approximately 45%.

These findings align with previous research. For instance, in [27], the authors investigated the damping properties of concrete with rubber waste additives and found that the inclusion of rubber waste decreased the dynamic modulus of elasticity while increasing the damping decrement of the concrete. This indicates enhanced energy dissipation capabilities, which corroborates the results obtained in this study.

Furthermore, in [28], the use of rubberized engineered cementitious composites in strengthening flexural concrete beams was explored. Their research highlighted that incorporating rubber particles improved energy dissipation and reduced wave propagation across concrete structures. This supports the observation that rubber-modified mortars can enhance the damping performance of concrete elements.

In terms of material selection, the hybrid use of SBR and EPDM granulates appears to offer a favorable balance between mechanical performance and vibroacoustic isolation. This observation is consistent with the findings of [29], in which the authors emphasized the potential of composite damping materials in optimizing vibration attenuation without excessive loss of stiffness.

In [30], the authors conducted an optimization of tire rubber–concrete core materials. This research focuses on optimizing rubber–concrete mixes for sandwich-structured composites, highlighting improvements in mechanical properties and thermo-acoustic insulation. The rubber–concrete mix demonstrated enhanced mechanical and insulation properties, making it suitable for use in lightweight, energy-efficient, and sustainable building materials.

The authors of [31] explored the use of crumb rubber as a replacement for sand in concrete, examining its impact on water absorption, compressive strength, and flexural strength. The authors demonstrated that replacing up to 10% of fine aggregate with crumb rubber did not significantly affect the compressive strength of concrete, and optimal flexural strength was achieved with a 5% replacement of fine aggregate with crumb rubber. Additionally, the authors noted that using crumb rubber as a replacement for fine aggregate resulted in cost savings and that the concrete product was environmentally friendly and did not harm nature.

The authors of [32] investigated cement mortar containing granulated rubber waste and brick fillers. The research results indicate that the introduction of rubber waste leads to a significant reduction in flexural strength, dynamic modulus of elasticity, and displacement. Brick waste can be considered a suitable filler that minimizes the negative impact of rubber and leads to the achievement of appropriate mixtures. Optimal values around 10% showed good agreement with experimental results, with differences not exceeding 5% for flexural strength and dynamic modulus of elasticity and 2% for displacement.

It is important to note that while the damping performance of rubberized layers was promising, the inclusion of granulates had a noticeable impact on compressive strength. This trade-off between mechanical and dynamic properties remains a key consideration for future design optimization. Additional research may focus on alternative binder systems, optimized particle gradation, or layered composite designs to further enhance the performance of such integrated solutions.

Moreover, this study focused on vertical vibration response, which is typically dominant in floor systems. Future investigations could expand the scope to include frequency-domain analysis, modal damping ratios, or fatigue performance under repetitive loads, which are critical factors in industrial environments.

## 5. Conclusions

This study presents an innovative approach to industrial flooring by integrating vibration-damping properties directly into the mortar layer using recycled rubber granulates. This technique not only overcomes the limitations of traditional vibration isolation systems but also promotes sustainability and multifunctional design. By analyzing various types and blends of rubber granulates, the research supports the development of advanced industrial floors that effectively balance structural performance with enhanced user comfort and adaptability to changing operational demands.

In the initial phase of the project, a reliable reference subbase concrete mix was successfully developed, meeting all technical and performance requirements, including workability, consistency, and compressive strength. The combination of ground granulated blast furnace cement, siliceous fly ash, and a polycarboxylate-based admixture ensured excellent rheological properties and long-term strength development. Additionally, the mix was designed with a strength reserve to offset the expected reduction in mechanical performance caused by the inclusion of vibration-isolating rubber granulates.

The research on the impact of various rubber granulate components on reducing vibration levels in industrial floors allows us to present the following key conclusions:

1. Functionality and safety are essential in high-demand environments, considering mechanical, chemical, and environmental factors. Concrete is strong and cost-effective, but evolving needs for better vibroacoustic performance drive innovation. Rubber granulates in cement layers improve vibration isolation but require balancing structural strength.
2. Integrating vibration-damping properties into mortar with recycled rubber granulates surpasses traditional systems, promoting sustainability and multifunctional design. Research supports developing advanced floors that balance structural performance with enhanced comfort and adaptability.
3. A reliable subbase concrete mix meeting technical and performance requirements was developed, combining ground granulated blast furnace cement, siliceous fly ash, and a polycarboxylate-based admixture. A strength reserve was included to counteract reduced mechanical performance from the rubber granulates.
4. Incorporating EPDM and SBR rubber granulates maintained good workability and self-compacting properties, even with 100% natural aggregate replacement. The suitability was validated through consistency and compressive strength tests, confirming vibration-damping performance.
5. Full-scale production and testing confirmed compliance with EN 206 [21] standards and project-specific requirements. Both mixes showed excellent workability, pumpability, and consistent performance. Compressive strength exceeded the required thresholds, demonstrating durability and reliability.
6. Mortars with EPDM and hybrid SBR–EPDM rubber granulates were produced at a ready-mix plant. Required flow properties were maintained, and target compressive strengths were achieved after curing, confirming technical feasibility and practical applicability.
7. Construction and dynamic testing of floor slabs confirmed feasibility and effectiveness. The mixes met the structural and dimensional requirements, with a significant reduction in vibration transmission, especially in the vertical direction.
8. The slab with hybrid EPDM/SBR rubber granulate mortar achieved the highest vibration-damping efficiency, reducing vertical acceleration by 58.6%. The EPDM-only mortar showed a significant reduction of 45.5%, highlighting its effectiveness as a sustainable alternative.
9. Incorporating rubber granulate, especially hybrid EPDM and SBR, significantly enhances vibration-damping performance and aligns with previous research showing improved energy dissipation and reduced wave propagation, improving vibroacoustic comfort.
10. Rubber-modified mortars show excellent damping but reduced compressive strength. Hybrid SBR and EPDM mixes offer a balance between mechanical integrity and vibration isolation. Future research should explore optimized designs, alternative binders, and layered systems, including frequency-domain analysis, modal damping, and fatigue behavior under cyclic loading.

## Figures and Tables

**Table 1:** Mix composition of the subbase concrete.

Component	Type/Description	Quantity [kg/m^3^]
Binder	CEM III/A 42.5 N	450
	Siliceous fly ash (Category A)	150
Water	-	250
Aggregate	Sand 0/2 mm	950
	Gravel 2/8 mm	475
Chemical admixture	Polycarboxylate ether (PCC)	3.8
Water-to-binder ratio (w/b)		0.50
Fresh concrete density [kg/m^3^]		2278
Sand point (percentage of fine aggregate) [%]		40.7

**Table 2:** Properties of the reference concrete mix—consistency and density.

Consistency				Density [kg/m^3^]
Slump Test Result [mm]		Flow Diameter [mm]		
After 10 min	After 60 min	After 10 min	After 60 min	
240	280	600	630	2260

**Table 3:** Compressive strength test results after 7 and 28 days.

No.	After 7 days			After 28 days			Concrete
	Failure Load F_i_ [kN]	Strength f_ci_ [N/mm^2^]	Mean Value f_cm_ [N/mm^2^]	Failure Load F_i_ [kN]	Strength f_ci_ [N/mm^2^]	Mean Value f_cm_ [N/mm^2^]	
1.	216	21.6	21.7	444	44.4	44.2	C25/30
2.	212	21.2		439	43.9		
3.	222	22.2		442	44.2		

**Table 4:** Consistency and viscosity class (t_500_) of concrete mixes with vibration-isolating rubber additives.

Additive Type	Dosage [% of Aggregate Fraction]	Slump [mm] After 10 min	Slump [mm] After 60 min	Viscosity Class (t_500_)
EPDM rubber granulates, 1/3 mm	30	220	260	VS1
	60	200	245	VS1
	100	210	265	VS1
SBR rubber granulates, 0.8/2 mm	30	180	200	VS1
	60	195	215	VS1
	100	185	200	VS1

**Table 5:** Average compressive strength of subbase concretes with vibration-isolating additives.

Additive Type	Dosage	Compressive Strength [MPa] After 7 days	Compressive Strength [MPa] After 28 days
EPDM rubber granulates, 1/3 mm	30	7.4	20.1
	60	5.8	18.4
	100	4.9	16.4
SBR rubber granulates, 0.8/2 mm	30	6.1	18.7
	60	5.4	15.4
	100	5.0	15.8

**Table 6:** Mix composition of self-compacting subbase concretes with 100% rubber aggregate replacement of natural aggregate.

Mix Symbol	Component	Quantity [kg/m^3^]
EPDM	CEM III/A 42.5 N	470
	Fly ash	375
	EPDM rubber granulates (1/3 mm)	410
	Water	425
	PCC admixture	5.0
SBR	CEM III/A 42.5 N	470
	Fly ash	378
	SBR rubber granulates (0.8/2 mm)	275
	Water	462
	PCC admixture	3.8
EPDM + SBR	CEM III/A 42.5 N	445
	Fly ash	365
	EPDM rubber granulates (1/3 mm)	175
	SBR rubber granulates (0.8/2 mm)	175
	Water	445
	PCC admixture	5.2

**Table 7:** Flowability and compressive strength of self-compacting subbase mixes with vibration-isolating additives.

Mix Type	Slump Flow [mm]		Compressive Strength				
			No.	After 7 Days		After 28 Days	
	After 10 min	After 60 min		Strength	Mean	Strength	Mean
				f_ci_ [N/mm^2^]	f_cm_ [N/mm^2^]	f_ci_ [N/mm^2^]	f_cm_ [N/mm^2^]
EPDM	280	290	1.	4.8	4.7	16.0	15.3
			2.	5.1		15.5	
			3.	4.2		14.3	
SBR	260	275	1.	3.2	3.6	12.5	13.1
			2.	3.8		13.8	
			3.	3.7		13.0	
EPDM + SBR	285	290	1.	5.2	5.2	17.2	16.5
			2.	5.4		15.9	
			3.	5.0		16.4	

**Table 8:** Rebound number results for composite specimens—the surface of the vibration-isolating layer.

Mixture	No.	Schmidt Rebound Value [-]									Mean Value [-]
		No.									
		1	2	3	4	5	6	7	8	9	
EPDM	1	24	26	26	25	26	24	27	26	25	25.48
	2	25	24	24	26	26	27	25	25	26	
	3	26	26	28	24	25	25	26	26	25	
SBR	1	30	28	30	29	29	28	29	28	29	29.29
	2	29	31	31	28	29	30	30	29	29	
	3	27	28	30	30	31	29	30	30	30	
EPDM + SBR	1	26	26	27	24	25	26	25	24	25	25.41
	2	25	26	26	25	24	27	25	25	26	
	3	26	28	25	25	25	26	24	25	25	

**Table 9:** Composition and selected parameters of C35/45 XM2 surface concrete.

Component Type	Component	Quantity [kg/m^3^] or [dm^3^/m^3^]
Binder	CEM III/A 42.5 N	400
	Fly ash	70
Water	–	180
Aggregate	Sand 0/2 mm	720
	Gravel 2/8 mm	340
	Gravel 8/16 mm	530
Chemical admixture	PCC (polycarboxylate ether)	4.95
Water/cement ratio		0.42
Fresh concrete density		2285 kg/m^3^
Sand point		41.6%
Paste volume		345 dm^3^/m^3^
Mortar volume		617 dm^3^/m^3^
Fine particles ≤ 0.125 mm		480 kg/m^3^

**Table 10:** Average compressive strength of surface concrete (C35/45 XM2) over time.

Curing time [days]	7	28	56	90	Concrete class (EN 206)
Strength [MPa]	29.2	49.6	51.6	56.2	C40/50
Standard deviation [±MPa]	2.1	2.9	2.8	2.9	

**Table 11:** Rheological properties and density of the lean subbase concrete mix.

Slump [mm]	Flow Spread [mm] (10 min)	Flow Spread [mm] (60 min)	Density [kg/m^3^]
255	610	635	2260

**Table 12:** Average compressive strength of lean subbase concrete over time.

Curing time [days]	7	28	56	90	Concrete class (EN 206)
Strength [MPa]	9.0	20.6	23.6	26.2	C16/20
Standard deviation [±MPa]	2.1	2.5	2.4	2.7	

**Table 13:** Mix composition of optimized vibration-isolating mortars with rubber granulates.

Mix Symbol	Component	Quantity [kg/m^3^]
EPDM	CEM III/A 42.5 N	450
	Fly ash	395
	EPDM rubber granulates (1/3 mm)	410
	Water	425
	PCC admixture	5.0
EPDM + SBR	CEM III/A 42.5 N	450
	Fly ash	375
	EPDM rubber granulates (1/3 mm)	175
	SBR rubber granulates (0.8/2 mm)	175
	Water	440
	PCC admixture	5.2

**Table 14:** Flow diameter and compressive strength of vibration-isolating mortars with EPDM and EPDM + SBR rubber granulates.

Mixture	Flow Diameter [mm]		Lp.	Compressive Strength					
				After 7 days			After 28 days		
	After 10 min	After 60 min		Strength f_ci_ [N/mm^2^]	Mean f_cm_ [N/mm^2^]	Standard Deviation [±N/mm^2^]	Strength f_ci_ [N/mm^2^]	Mean f_cm_ [N/mm^2^]	Standard Deviation [±N/mm^2^]
EPDM	285	290	1.	4.5	4.7		14.8		0.25
			2.	4.8		0.15	15.0	15.0	
			3.	4.7			15.3		
EPDM + SBR	280	295	1.	5.5	5.5		18.4		0.50
			2.	5.9		0.40	17.8	17.9	
			3.	5.1			17.4		

**Table 15:** Maximum accelerations for each floor.

No.	A_X	A_Y	A_Z	B_X	B_Y	B_Z	C_X	C_Y	C_Z
1	13.72	9.55	61.59	15.26	4.25	28.52	8.70	4.04	32.99
2	16.79	9.84	61.45	11.35	4.87	22.05	9.05	5.75	33.50
3	18.51	9.75	62.23	12.12	6.31	26.20	10.37	6.81	34.50
Mean	16.34	9.71	61.76	12.91	5.14	25.59	9.37	5.53	33.66
Standard deviation	2.43	0.15	0.42	2.07	1.06	3.28	0.88	1.40	0.77

## Data Availability

The original contributions presented in the study are included in the article, further inquiries can be directed to the corresponding author.
